# A Case of Ameloblastic Fibroodontoma Extending Maxillary Sinus with Erupted Tooth: Is Transcanine Approach with Alveolectomy Feasible?

**DOI:** 10.1155/2016/8594074

**Published:** 2016-11-06

**Authors:** Mustafa Aslıer, Mustafa Cenk Ecevit, Sülen Sarıoğlu, Semih Sütay

**Affiliations:** ^1^Department of Otorhinolaryngology, Dokuz Eylul University School of Medicine, Izmir, Turkey; ^2^Department of Pathology, Dokuz Eylul University School of Medicine, Izmir, Turkey

## Abstract

Ameloblastic fibroodontoma (AFO) is a rare entity of mixed odontogenic tumors and frequently arises from posterior portion of the maxilla or mandible in first two decades of life. Herein, a 35-year-old woman with a noncontributory medical history who presented with a progressive left maxillary toothache, left maxillary first molar tooth mobility, and swelling in the left maxillary molar area for the last 2 months was reported. Radiologically, a tumor that originated from periapical area of the second mature molar teeth of maxilla was seen and additively unerupted tooth was not detected. The histopathologic examination revealed AFO. The patient is disease-free for five years after treated with limited segmental alveolectomy combining with Caldwell-Luc procedure.

## 1. Introduction

Ameloblastic fibroodontoma (AFO) is an uncommon mixed odontogenic tumor of odontogenic epithelium and mesenchyme origin [1, 2]. According to the latest World Health Organization (WHO) classification, AFO is a lesion resembling ameloblastic fibroma which also shows inductive alterations composed of both enamel and dentin [3]. Typical features of AFO show a slow growth swelling from posterior portion of the maxilla or mandible generally in the first and rarely in the second decades of life. It is generally associated with unerupted teeth of the affected area. Radiological appearance shows a well-defined radiolucent border with radiopaque foci containing density similar to that of dental hard tissues [4, 5]. Conservative enucleation or curettage is enough for adequate treatment of AFO and radical surgical procedures such as segmental resection or hemimandibulectomy are infrequently needed [5, 6]. Here in, a case of AFO arising from the posterior portion of the maxilla of a middle-aged female was presented.

## 2. Case Report

**Figure 1:**
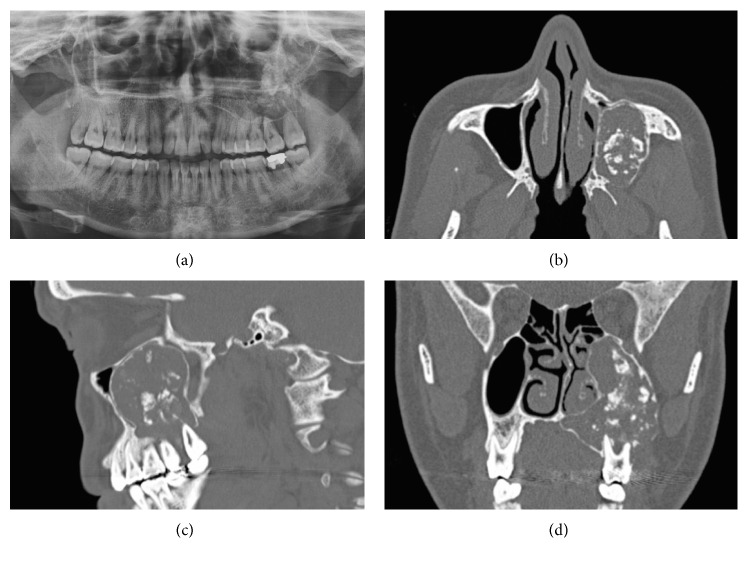
(a) Preoperative OPR showing a radiopaque mass with a radiolucent border in the left upper jaw. (b, c, d) Preoperative CTI showing a radiolucent expansive lesion containing multiple radiopaque foci on the left side maxillary sinus.

**Figure 2:**
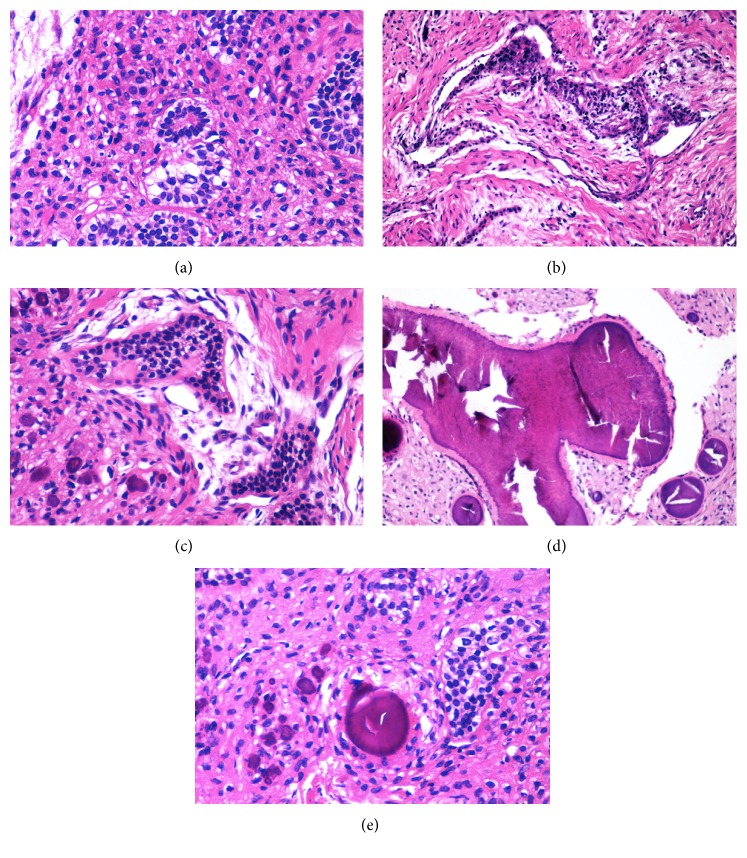
(a) Ameloblastic epithelium in the fibroblastic stroma (H&E ×20). (b) Odontogenic epithelium forming premature tooth like pattern (H&E ×10). (c) Odontogenic epithelium forming material consistent with dentine (H&E ×10). (d) Calcific zones surrounded with dentine (H&E ×10). (e) Focal zones consistent with cementifying changes (H&E ×20).

**Figure 3:**
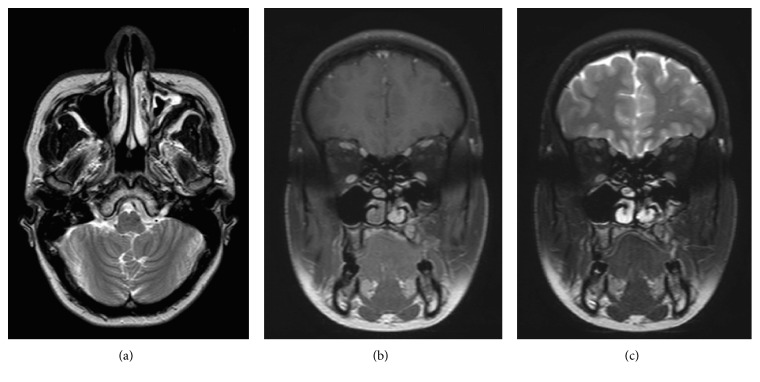
(a, b, c) Respectively, axial T2, coronal T1, and coronal T2 postoperative MRI showing mucosal thickening in the maxillary without any evidence of tumor recurrence.

A 35-year-old female presented with a 2-month history of progressive left maxillary toothache, left maxillary first molar tooth mobility, and swelling in the left maxillary molar area. There was no history of chronic nasal problems or odontogenic surgery. Swelling on the left side of the maxillary alveolar arch was revealed by intraoral inspection. The rest of the ENT examination was unremarkable. Preoperative oral panoramic radiograph (OPR) and computer tomography imaging (CTI) of the paranasal sinus revealed a radiolucent expansive lesion containing multiple radiopaque foci on the left side maxillary sinus (Figure 1). An incisional biopsy was performed through canine fossae approach. The histopathological examination revealed fibroblastic connective tissue matrix containing ameloblastic and odontogenic epithelial component was shown on the histopathological examination. After completion of diagnostic investigation, curative resection was approved. Under general anaesthesia with transnasal intubation, five-centimetre mucosal incision was made in the gingivolabial sulcus, extending from the canine tooth to the first molar tooth. Soft tissue and periosteum were elevated and four-centimetre diameter fenestration was made on the anterior wall of the maxillary sinus. Thereafter, tumor was dissected quite easily from the anterior, medial, and superior walls of the sinus. Posterior dissection was hardly performed because of bleeding. Next, all of the three molar teeth of maxilla were extracted and tumor resection was completed combining with limited segmental alveolectomy. Tumor originated from the second molar teeth of maxilla and progressed into maxillary sinus was detected when the specimen was examined. At the final stage, mucosal incisions were then closed using absorbable sutures. Postoperative healing was uneventful. Definitive pathology report was similar to the previously examined and tumor was diagnosed as AFO (Figure 2). The patient remains clinical and radiological disease-free up till a five-year follow-up period. Postoperative magnetic resonance imaging (MRI) revealed mucosal thickening in the maxillary without any evidence of tumor recurrence (Figure 3).

## 3. Discussion

Ameloblastic fibroodontoma is a rare entity of mixed odontogenic tumors and frequently arises from posterior portion of the maxilla or mandible in first two decades of life [1, 7, 8]. But as it is seen in our case, it may be presented at middle age period. Histologically, the WHO classification defines AFO as “a lesion similar to ameloblastic fibroma (AF), but also showing inductive changes that lead to formation of both dentin and enamel” [3]. Most investigators suggested that the age of the patient was the essential distinction between AF and AFO [1]. However, pathological examination combining immunohistochemical staining shows important evidences of AFO [2, 7]. In the present study, histopathologically ameloblastic and odontoid ectomesenchymal tissues including dentin-like calcified structures in fibroid stroma were seen and according to these findings the final diagnosis was AFO in a 35-year-old female.

Asymptomatic slow growth swelling and delayed tooth eruptions are the most common symptoms of AFO especially at early ages [2, 8, 9]. The tumor is predominantly related unerupted tooth but at times it may arise from a supernumerary or deciduous tooth [10]. The patient presented in this report had no unerupted tooth. According to this feature AFO may occur after the tooth eruption process completed. Therefore, tooth problems like toothache and tooth mobility may also be included to the signs and symptoms of AFO at advanced ages.

Radiological findings of the AFO were described in the previous studies. Dental like radiopaque foci surrounding well-defined radiolucent borders are the most detectable feature of AFO in radiological examinations [4, 5]. Radiological findings of the AFO were similar in the present patient. Preoperative oral panoramic radiograph and CTI showed radiopaque foci containing multiple dental like densities surrounding radiolucent area and sclerotic border which were suggesting AFO (Figure 1).

The curative treatment of AFO involves enucleation or curettage. These approaches are frequently sufficient if the lesion is associated with unerupted teeth and the affected area is limited. In a small group of patients who have giant, extensive, and destructive disease, partial maxillectomy or segmental mandibulectomy may be needed [1, 2]. But even AFO is believed to have low potential for recurrence; according to Boxberger, almost all cases of recurrences were related to incomplete removal of the lesion at the initial surgery [11]. In this report, lesion was originated from the periapical area of the left maxillary second molar tooth and grown into the maxillary sinus. Anterior, superior, and medial walls of the sinus were preserved. By the reasons of these features, partial maxillectomy was found super abound and unnecessary. Limited segmental alveolectomy combining with Caldwell-Luc procedure was done and the patient was disease-free for five years. We suggest combination of trans-canine-fossa approach with segmental alveolectomy as an uncomplicated, safe, and feasible approach for AFO extending to the maxillary sinus.

In conclusion, AFO may also occur at advanced ages with odontogenic symptoms and the extension of lesion manages the curative treatment alternatives.
